# Supplementary material for: Crystal structures of bacterial small multidrug resistance transporter EmrE in complex with structurally diverse substrates
Source: eLife. 2022 Mar 7;11:e76766. doi: 10.7554/eLife.76766 (PMC9000954; doi:10.7554/eLife.76766)
Supplement: Transparent reporting form [file elife-76766-transrepform1.docx]

***eLife’s* transparent reporting form**

We encourage authors to provide detailed information *within their submission* to facilitate the interpretation and replication of experiments. Authors can upload supporting documentation to indicate the use of appropriate reporting guidelines for health-related research (see [EQUATOR Network](http://www.equator-network.org/%20)), life science research (see the [BioSharing Information Resource](https://biosharing.org/)), or the [ARRIVE guidelines](http://www.plosbiology.org/article/info:doi/10.1371/journal.pbio.1000412) for reporting work involving animal research. Where applicable, authors should refer to any relevant reporting standards documents in this form.

If you have any questions, please consult our Journal Policies and/or contact us: [editorial@elifesciences.org](mailto:editorial@elifesciences.org).

**Sample-size estimation**

- You should state whether an appropriate sample size was computed when the study was being designed
- You should state the statistical method of sample size computation and any required assumptions
- If no explicit power analysis was used, you should describe how you decided what sample (replicate) size (number) to use

Please outline where this information can be found within the submission (e.g., sections or figure legends), or explain why this information doesn’t apply to your submission:

No statistical analysis other than standard error of measurement was used to show variance between replicates, so no sample size pre-determination was required. At least n=3 independent experiments were performed for each mutant, which is standard for biochemical assays.

**Replicates**

- You should report how often each experiment was performed
- You should include a definition of biological versus technical replication
- The data obtained should be provided and sufficient information should be provided to indicate the number of independent biological and/or technical replicates
- If you encountered any outliers, you should describe how these were handled
- Criteria for exclusion/inclusion of data should be clearly stated
- High-throughput sequence data should be uploaded before submission, with a private link for reviewers provided (these are available from both GEO and ArrayExpress)

Please outline where this information can be found within the submission (e.g., sections or figure legends), or explain why this information doesn’t apply to your submission:

Data were reproduced in multiple, independent experiments.

SSM electrophysiology: For each protein/substrate pair, full Michaelis Menten titrations (2-3 perfusions for each concentration) were collected from 4-6 independent sensor preparations. These, in turn, were prepared with protein from two-three independent protein preparations (biological replicates). Michaelis-Menten fits to each dataset were performed independently; Km values for the independent fits are reported in Figure 1-Figure Supplement 2. This is described in the legends of Figure 1 and Figure 1-Figure Supplement 2. For the monobody binding experiment, independent perfusion series were performed with three independently prepared sensors from three independent biochemical preps of EmrE3 and two independent biochemical preps of monobody L10(biological replicates). For mutant analysis with SSM electrophysiology: Traces shown are representative of three independent experiments (different sensors) performed with samples from each of two different biochemical preps. Peak currents for all replicates are reported in Table 2. All attempts at replication were successful.

Exclusions: If currents differed by more than 10% between the first and last perfusions, this indicated that the proteoliposomes associated with the sensor had not remained stable over the course of the experiment, and data collected in this series was discarded. Criteria were pre-determined. This is described in the Methods section.

Outliers: None

Microscale thermophoresis: Experiments were performed with three independently prepared samples for each concentration point (except for the 3 and 10 uM points, for which there are 2 replicates due to accidental sample loss). The mean and SEM of these measurements are reported in Figure 1c. This is described in the leged of Figure 1c. Since the observation of monobody binding was validated through other experimental methods (crystallography), only one protein prep was done. No data was excluded. We did not remove any outliers.

Information on the number of replicates for each experiment can be found in the figure legends.

**Statistical reporting**

- Statistical analysis methods should be described and justified
- Raw data should be presented in figures whenever informative to do so (typically when N per group is less than 10)
- For each experiment, you should identify the statistical tests used, exact values of N, definitions of center, methods of multiple test correction, and dispersion and precision measures (e.g., mean, median, SD, SEM, confidence intervals; and, for the major substantive results, a measure of effect size (e.g., Pearson's r, Cohen's d)
- Report exact p-values wherever possible alongside the summary statistics and 95% confidence intervals. These should be reported for all key questions and not only when the p-value is less than 0.05.

Please outline where this information can be found within the submission (e.g., sections or figure legends), or explain why this information doesn’t apply to your submission:

Data is reported as the mean and SEM of independent measurements. This information can be found in the associated figure legends.

(For large datasets, or papers with a very large number of statistical tests, you may upload a single table file with tests, Ns, etc., with reference to sections in the manuscript.)

**Group allocation**

- Indicate how samples were allocated into experimental groups (in the case of clinical studies, please specify allocation to treatment method); if randomization was used, please also state if restricted randomization was applied
- Indicate if masking was used during group allocation, data collection and/or data analysis

Please outline where this information can be found within the submission (e.g., sections or figure legends), or explain why this information doesn’t apply to your submission:

Samples were not allocated into groups for data collection or analysis since standard error of measurement was the only statistical test used to show variance between replicates

**Additional data files (“source data”)**

- We encourage you to upload relevant additional data files, such as numerical data that are represented as a graph in a figure, or as a summary table
- Where provided, these should be in the most useful format, and they can be uploaded as “Source data” files linked to a main figure or table
- Include model definition files including the full list of parameters used
- Include code used for data analysis (e.g., R, MatLab)
- Avoid stating that data files are “available upon request”

Please indicate the figures or tables for which source data files have been provided:

1b, 1c, 1d, 1e, 1-S2, 5, Table 2.

Mtz and pdb files for crystal structures.
